# Editorial: Engineered medicines to mitigate resistance to cancer immunotherapy

**DOI:** 10.3389/fmed.2024.1452812

**Published:** 2024-08-06

**Authors:** Kishu Ranjan, Prabhatchandra Dube, Sandeep Kumar Mishra

**Affiliations:** ^1^Department of Pathology, Yale University, New Haven, CT, United States; ^2^Department of Medicine, University of Toledo College of Medicine and Life Sciences, Toledo, OH, United States; ^3^Department of Radiology and Biomedical Imaging, Yale University, New Haven, CT, United States

**Keywords:** cancer immunotherapy, tumor immunology, engineered medicines, CAR-T cells, personalized therapy

Cancer immunotherapy elicits durable objective responses in patients affected by a variety of cancers. Immunotherapies, which leverage and enhance patients' immune system, encompass antibodies that inhibit suppressive immune-checkpoint pathways, cellular therapies utilizing dendritic cells and engineered T cells, as well as vaccines that elicit antigen-specific immune responses in tumors (1, 2). Despite the substantial response rates observed in cancer immunotherapies, the issue of resistance to immunotherapy continues to be a multifaceted clinical problem affecting a significant proportion of patients (3). Moreover, immunotherapies can lead to the development of various organ-specific immune-related adverse events (irAEs) due to their mechanism of action. Nonetheless, significant advancements have been made in comprehending the factors that contribute to these toxicities, as well as implementing effective strategies for their management (4). Various strategies have been implemented to address resistance to immunotherapy, including personalized therapy and the development of engineered medicines. These approaches are based on the principle that the patient's immune cells exhibit no cross-reactivity, thereby enabling the engineering of a sustainable antitumor immune response.

Personalized therapy and engineered medicine revolutionize cancer immunotherapy by tailoring treatments to individual patients' genetic profiles. Advanced techniques, like CAR-T cell therapy, reprogram patients' immune cells to target cancer cells specifically. This approach enhances efficacy and minimizes side effects, offering a promising future for precise, patient-specific cancer treatment (3, 5).

This Research Topic aims to emphasize the importance of immunotherapies targeting tumor progression, and strategies for the management of toxicities associated with immunotherapies. Our ultimate objective is to share recent advancements in the field of immunotherapy by uncovering modern innovative tools and approaches that can contribute to achieving long-lasting clinical outcomes.

Chimeric antigen receptor (CAR) T-cell therapy is a personalized cell-based therapy that harnesses the patient's own immune cells to target and eradicate cancer cells with high precision and efficacy. Chen et al. comprehensively discussed the development of next-generation approaches aimed at improving the effectiveness of allogeneic (donor-derived) cell-based cancer therapies, major challenges, and future directions. The authors drew attention to several prospective investigations aimed at genetically augmenting antigen recognition and binding, as well as manipulating transcription factors and the epigenetic regulation of CAR immune cells. Overall, allogeneic immune cells possess the advantage of minimal alloreactivity, while also offering the capacity for genetic modification to heighten anti-tumor reactivity and achieve enduring treatment efficacy.

Jungen et al. explored the spatial distribution patterns of CD3^+^ and CD8^+^ lymphocytes as a potential pretest method to identify POLE wild-type molecular subgroups in endometrial carcinoma (EC). The authors employed a multiplex immunofluorescence assay to quantitatively examine the spatial arrangement or distribution patterns of CD3^+^ and CD8^+^ cells within the tumor microenvironment of EC. The results revealed that an intra-tumoral CD8^+^ lymphocyte density of less than 50/mm^2^ is the most predictive value for considering a POLE wild-type condition. In conclusion, this study has potential implications for molecular subtyping of the disease, with the aim of enhancing diagnostic accuracy and informing treatment decisions in the clinical setting.

In our Research Topic in the Journal, Hu et al., summarize the effectiveness and safety of CAR-T treatment for patients with relapsed/refractory multiple myeloma (RRMM). RRMM has remained an incurable condition despite significant advancements in therapies over the past few decades. CAR-T treatment represents cutting-edge approaches, has shown to be an effective therapy for personalized clinical cancer immunotherapy. This meta-analysis demonstrated that anti-BCMA CAR-T therapy provided significant benefits with an acceptable safety profile in patients with RRMM. However, despite its impressive efficacy, the associated toxicities post-treatment has restricted the broader adoption of anti-BCMA CAR-T therapy for multiple myeloma. The study comprehensively described the structure of CAR-T products, examined how CAR-T composition affects outcome indicators, and conducted a subgroup analysis in RRMM patients undergoing CAR-T therapy. Additionally, the study systematically evaluated eighteen efficacy and safety outcome measures for anti-BCMA CAR-T therapy for multiple myeloma. The findings of this study provide guidance on the timing of intervention for systemic inflammatory reaction toxicity following CAR-T cell infusion, helping to prevent serious complications.

Mowforth et al., reported a systematic review on the survival efficacy of personalized therapies in glioblastoma. Glioblastoma (GBM), the prevalent and aggressive primary brain tumor, yields a median survival of 15 months and a 5-year survival rate of merely 5%, defying curative efforts due to its extensive infiltration beyond surgical and radiological boundaries. A promising approach lies in personalized medicine, which aims to improve survival by targeting individualized patient characteristics. This represents a paradigm shift from disease-centric treatments that overlook variations between patients, moving instead toward precise and individualized therapies that address each patient's unique attributes. This systematic review included more than 20 types of personalized therapy and found that targeted molecular therapies were the most studied (33.3%), followed by autologous dendritic cell vaccines (32.4%) and autologous tumor vaccines (10.8%). Personalized glioblastoma therapies remain of unproven survival benefit.

Gillette et al. explored the unique challenges of GBM, focusing on both intrinsic and adaptive immune resistance. It highlights the limited success of current immunotherapies and explores various engineered and non-engineered treatments designed to overcome GBM resistance. The review addressed key barriers to the success of immunotherapies, such as the tumor's immunosuppressive microenvironment, genetic heterogeneity, and adaptive resistance mechanisms. It also explored strategies to overcome these challenges, highlighting promising combinatorial approaches, like combining immunotherapy with maximal resection, radiation, and chemotherapy to enhance treatment efficacy. The goal is to identify approaches that can improve patient prognosis and clinical outcomes, emphasizing the need for continued research to develop effective GBM-targeted immunotherapies.

An insightful opinion article by Adamo et al., emphasizes the importance of educating professionals to enhance the efficient translation of Advanced Therapy Medicinal Products (ATMPs) from bench to bedside. They present the view that new personalized treatments, particularly cell and gene therapies, offer significant advantages by precisely targeting biological mechanisms to repair and restore tissues. These therapies, including successful examples like CAR-T cell therapies for certain cancers, are regulated in the European Union (EU) as ATMPs. However, their complexity often hinders their transition from research to clinical practice due to regulatory, manufacturing, and clinical challenges. Demonstrating safety and efficacy is crucial, given the invasive nature of these treatments. Education and early collaboration among scientists, regulatory bodies, and clinicians are essential to overcoming these obstacles. Long-term follow-up and data sharing are vital for understanding these therapies and securing regulatory approval, advancing the development and application of ATMPs.

Overall, this compilation demonstrates the collaborative efforts of researchers who are eagerly striving to design effective anti-tumor immunotherapies and to improve patients' lives.

## Author contributions

KR: Conceptualization, Writing – original draft, Writing – review & editing. PD: Conceptualization, Writing – original draft, Writing – review & editing. SKM: Conceptualization, Writing – original draft, Writing – review & editing.
